# Exacerbation of Hyperbilirubinemia by Falciparum Malaria in a Patient With Coexisting Gilbert’s Syndrome and Glucose-6-Phosphate Dehydrogenase Deficiency

**DOI:** 10.7759/cureus.73073

**Published:** 2024-11-05

**Authors:** Hsu Y Mon, Helen Alemayehu, Keerthana Pampapathi, Samson O Oyibo

**Affiliations:** 1 Medicine, Peterborough City Hospital, Peterborough, GBR; 2 Diabetes and Endocrinology, Peterborough City Hospital, Peterborough, GBR

**Keywords:** blood hemolysis, falciparum malaria, gilbert’s syndrome, glucose-6-phosphate-dehydrogenase deficiency (g6pd), unconjugated hyperbilirubinemia

## Abstract

Gilbert’s syndrome and G6PD deficiency are common genetic disorders. They both give rise to unconjugated hyperbilirubinemia through different mechanisms. Falciparum malaria-induced hemolysis is another cause of unconjugated hyperbilirubinemia. We have reported a 51-year-old Asian male who presented with a four-day history of fever and sweating just after a holiday in Kenya. He admitted to not taken malaria prophylaxis while on holiday. Initial investigations revealed that he had falciparum malaria along with co-existing severe G6PD deficiency and Gilbert’s syndrome, which we believe, all contributed to an exacerbation of unconjugated hyperbilirubinemia (four-fold rise). He was treated with intravenous fluids, paracetamol and oral artemether/lumefantrine combination therapy. He made a good clinical recovery. After a month, he still exhibited chronic unconjugated hyperbilirubinemia with recurrent elevation in his reticulocyte count but no anaemia, suggesting further episodes of compensated hemolysis. We also discuss the differential diagnosis for unconjugated hyperbilirubinemia in relation to this interesting case.

## Introduction

Hyperbilirubinemia is a condition defined as elevated serum bilirubin levels above the reference range. Hyperbilirubinemia can be unconjugated (indirect) or conjugated (direct) [1]. Bilirubin is a product of heme breakdown. It is bound to albumin in its unconjugated form before being presented to the liver to get conjugated with glucuronic acid by the enzyme uridine diphospho-glucuronate glucuronosyltransferase-1A1 (UGT1A1). This process makes bilirubin soluble so that it is easily excreted and less likely to diffuse across the blood-brain barrier [1]. Therefore, unconjugated hyperbilirubinemia can result from increased production from increased heme breakdown (e.g., hemolysis, dyserythropoiesis), impaired liver uptake of bilirubin (e.g., liver congestion, portosystemic shunt, drugs, etc.) and impaired conjugation of bilirubin due to genetic mutations in the UGT1A1 gene (e.g., Gilbert’s syndrome, Crigler-Najjar syndrome) [1]. In newborns, unconjugated hyperbilirubinemia can cause bilirubin-induced encephalopathy due to unconjugated bilirubin crossing the blood-brain barrier. In adults, it can cause jaundice, pale stools, itchy skin, weakness, and poor appetite.

Gilbert’s syndrome is an autosomal recessive liver disorder where there is reduced glucuronidation of bilirubin with resultant unconjugated hyperbilirubinemia and recurrent jaundice. The UGT1A1 gene is located on the long arm of chromosome-2 (2q37), and in the majority of cases, there is a dinucleotide insertion (thymine-adenine, TA) in the promoter region of the UGT1A1 gene resulting in seven copies (7TA repeats) instead of the usual six [2]. Gilbert’s syndrome has a prevalence rate of 4-16%, and this can vary depending on ethnic ancestry; that is 2-10% in the White population, 2% in Japan and East Asia and up to 20% in India, Southern Asia, and the Middle East [2]. Episodes of hyperbilirubinemia can be precipitated by fasting, hemolytic reactions, febrile illnesses, menstruation, and physical exertion. Gilbert syndrome does not require treatment; however, it must be distinguished from other diseases that also cause unconjugated hyperbilirubinemia [2].

Glucose-6-phosphate dehydrogenase (G6PD) deficiency is a genetic disorder of red blood cells that affects 400 million people worldwide. It is most prevalent in tropical and subtropical areas, and particularly common in people of African, Mediterranean, or Asian descent [3,4]. G6PD is an enzyme that converts nicotinamide adenine dinucleotide phosphate to its reduced form, which is essential for red blood cell protection against oxidative stress. Without this protection, red blood cells become prone to oxidative stress-induced hemolysis caused by numerous drugs, chemicals, food substances, infections, and stress. G6PD deficiency is due to a mutation in the G6PD gene, which is located on the long arm of the X-chromosome (Xq28). Therefore, transmission is by X-chromosome recessive inheritance and thus, it affects hemizygous males and the rare homozygous females [4,5]. The residual enzyme activity varies with different variants and is usually enough such that individuals are asymptomatic unless provoked by known stressors. Severe deficiency with less than 10% residual activity can result in chronic non-spherocytic hemolytic anemia, and it can lead to chronic unconjugated hyperbilirubinemia. G6PD deficiency is common in malaria-endemic areas, and some antimalarial drugs are known stressors. Therefore, it is important to test patients for G6PD deficiency before starting antimalarial therapy [4,5]. If a male patient is suspected of having G6PD deficiency on clinical grounds, either the fluorescent spot or the dye decolourisation screening test are acceptable first-line tests. If the screening test is abnormal or equivocal, the quantitative assay should be undertaken to confirm, or exclude, the diagnosis [4,5].

Malaria is a parasitic infection transmitted by the female Anopheles mosquito. It is an acute life-threatening disease and poses a significant global health threat. Malaria endemic areas include Western and sub-Saharan Africa, South Asia, the Western Pacific, and Central America [6]. The life cycle of the malaria parasite (Plasmodium) involves rapid division, multiplication, and invasion of the liver cells and red blood cells. After an incubation period of 7-14 days, most infected individuals present with fever, headaches, malaise, weakness, and gastrointestinal symptoms. Prompt treatment is necessary, as delayed treatment can result in severe hemolytic anemia, hepatopathy, cerebral malaria, coma, and death [6]. The features of severe or complicated malaria include impaired consciousness, convulsions, renal impairment, acidosis, hypoglycemia, respiratory distress, severe anemia, shock, spontaneous bleeding, hemoglobinuria, and >5% parasitemia [7]. Artemether-lumefantrine combination therapy is the first-line choice for the treatment of uncomplicated falciparum malaria acquired in areas with chloroquine resistance [8]. Severe or complicated falciparum malaria requires initial treatment with intravenous artesunate in a high-dependency unit or intensive care setting, followed by artemisinin combination therapy [8].

We report a patient who was discovered to have Gilbert’s syndrome and G6PD deficiency during a hospital admission for falciparum malaria. We also discuss the differential diagnosis of unconjugated hyperbilirubinemia in relation to this case.

## Case presentation

Medical history and demographics

A 51-year-old Asian male presented to the emergency department with a four-day history of fever and sweating, following a recent travel to Kenya. He had been on a three-week holiday in Kenya (Mombasa during the first week, Nairobi during the second week, and back in Mombasa during the third week). During the second week, he had a four-day bout of vomiting and diarrhea, which after three negative tests for malaria parasite, was treated with a five-day course of oral amoxicillin. He recovered and felt well for the next five days while back in Mombasa. However, two days before leaving Kenya back to the United Kingdom he developed the fever and sweating. On review of systems, he also had a mild headache, dizziness, and a non-productive cough. There was no complaint of photophobia or neck stiffness, and no complaint of hematemesis, bloody stools or frank hematuria. He did mention having a single episode of dark urine the day before presenting. His past medical history included ulcerative colitis for which he had curative surgery 16 years ago, and chronic mild hyperbilirubinemia for 17 years (baseline serum bilirubin ≤38 µmol/L). The hyperbilirubinemia had been extensively investigated in the past (e.g., normal liver antibodies, hepatitis screen, alpha-fetoprotein levels). An ultrasound scan at that time demonstrated mild fatty infiltration only. He also mentioned that he had an episode of malaria during childhood but could not remember specific details. His drug history included a recent course of amoxicillin for the vomiting and diarrhea while in Kenya, and cinnarizine for the dizziness. Regular medication included lansoprazole 30 mg daily and atorvastatin 40 mg daily. He admitted that he did not take malaria prophylaxis while he was in Kenya. He was allergic to penicillin. He was a non-smoker and did not drink alcohol.

On examination, he was conscious and alert with a Glasgow Coma Scale of 15 out of 15. He had a temperature of 36.2ºC, a heart rate of 76 beats per minute, a respiratory rate of 20 breaths per minute, a blood pressure of 113/66 mmHg, and his oxygen saturation was 96% on room air. He weighed 79 kg. He had conjunctival pallor, and his sclera revealed jaundice. His chest was clear, and his abdomen was soft and non-tender with no palpable organomegaly. There were no stigmata of chronic liver disease. A full neurological examination did not reveal any signs of meningeal irritation or cerebral dysfunction.

Investigations

At initial presentation, his hemoglobin and white cell count were normal, C-reactive protein was elevated, and there was mild thrombocytopenia with a slightly elevated prothrombin time. His serum bilirubin, reticulocyte count, and serum lactate dehydrogenase levels were elevated, indicating hemolysis (Table 1). A malaria parasite antigen test was positive. A blood film examination demonstrated significant plasmodium falciparum parasitemia. A G6PD fluorescent spot test was positive for G6PD deficiency, and a G6PD spectrometry confirmed an incredibly low G6PD enzyme activity (<10% of the normal range). Liver and kidney function tests were all normal. Tests for other hemoglobinopathies were negative. Blood cultures revealed no bacterial or fungal growth. Historical liver function test results demonstrated mild hyperbilirubinemia with a baseline ≤38 µmol/L for 17 years. Subsequent genetic analysis for Gilbert’s syndrome revealed that he was homozygous for seven thymine-adenine (7TA) repeats in the promoter region of the UGT1A1 gene, consistent with a diagnosis of Gilbert’s syndrome.

**Table 1:** Initial blood test results Results demonstrated hyperbilirubinemia, elevated reticulocyte count, elevated lactate dehydrogenase level and G6DP deficiency. The blood film was positive for Plasmodium falciparum parasites. *Percentage parasitemia is the percentage of a fixed number of red blood cells that are parasitized.

Blood test	Value	Reference Range
White Cell Count (10^9^/L)	7.3	4.0-11.0
Hemoglobin (g/L)	145	130-180
Reticulocyte count (10^9^/L)	121	23-81
Platelets (10^9^/L)	121	150-400
Sodium (mmol/L)	133	133-146
Potassium (mmol/L)	3.7	3.5-5.3
Chloride (mmol/L)	96	95-108
Creatinine (µmol/L)	98	59-104
Total bilirubin (µmol/L)	130	0-21
Conjugated bilirubin (µmol/L)	17	<7
Unconjugated bilirubin (µmol/L)	113	3-13
Alanine transferase (IU/L)	31	<41
Total Protein (g/L)	68	60-80
Globulin (g/L)	29	20-30
Amylase (IU/L)	83	<100
Albumin (g/L)	39	35-50
Alkaline phosphatase (IU/L)	81	30-130
Aspartate transferase (IU/L)	41	10-50
Lactate (mmol/L)	4	0.6-2.5
Prothrombin time (seconds)	16.9	9.4-16.4
Activate plasma thromboplastin time (seconds)	31.6	24-36
Fibrinogen (g/L)	5	1.7-4.5
Glucose (mmol/L)	6.4	<7.0
Glycated hemoglobin (mmol/mol)	27	120-42
C-reactive protein (mg/L)	98	<5
Lactate dehydrogenase (U/L)	298	<250
Plasmodium species antigen test	Plasmodium falciparum	Negative
Percentage parasitemia (%)*	1.97	0
G6DP screening test	Deficient	Normal
G6DP activity (U/g hemoglobin)	0.6	6.0-6.13

Treatment

The patient did not have any features of severe or complicated malaria, so therefore treated as uncomplicated falciparum malaria. The patient was initially treated with paracetamol and intravenous fluids. After a discussion with the microbiologist regarding the diagnosis of falciparum malaria and G6PD deficiency, the patient was given a 60-hour course of oral artemether/lumefantrine (20mg/120mg) combination therapy (an initial dose of four tablets, followed by four tablets at 8, 24, 36, 48, and 60 hours). An intravenous glucose-saline infusion was given because of therapy-induced nausea and poor appetite. Blood glucose values were within the normal range throughout treatment.

Outcome and follow-up

After starting antimalarial drugs, the patient’s condition improved, and he was discharged home on day-4. Malaria parasite count fell to 0.21% on day-2 and there was complete clearance on repeat blood film examination on day-6. His reticulocyte count fell to normal levels on day-2 but counts were elevated again on day-31 and day-55 after initiation of antimalarial therapy, indicating compensated hemolysis. His bilirubin levels were near to baseline (Table 2). The patient was subsequently commenced on folic acid 5 mg daily and given patient information leaflets about G6PD deficiency and Gilbert's syndrome. He was also advised to take malaria prophylaxis when travelling to malaria-endemic countries. He continued outpatient follow-up with the hematologist.

**Table 2:** Blood results during and after recovery *An unconjugated bilirubin fraction of 33 µmol/L (normal range: 3-13) indicates unconjugated hyperbilirubinemia.

Blood test	Days after initiation of antimalarial therapy				Normal values
	Day-2	Day-7	Day-31	Day-55	
Hemoglobin (g/L)	106	119	138	147	130-180
White blood cells (10^9^)	4.7	5.3	4.6	4.9	4.0-11.0
Reticulocyte count (10^9^)	50	-	105	84	23-81
Platelet count (10^9^)	50	199	190	202	150-400
Bilirubin (µmol/L)	59	-	41	42*	0-21
Lactate dehydrogenase (U/L)	289	-	198	178	<250
Percentage parasitemia (%)	0.21	Nil	Nil	Nil	Nil

## Discussion

We have reported a middle-aged man who presented with fever and sweating, which started while on holiday in Kenya and was discovered to have falciparum malaria on a background of severe G6PD deficiency and Gilbert’s syndrome, which all probably contributed to an exacerbation of unconjugated hyperbilirubinemia. His hemoglobin level was normal on admission, but his reticulocyte count and serum bilirubin levels were high. His hemoglobin level fell to anemic levels on the second day after commencing antimalarial therapy. He made clinical recovery by the fourth day, and his full blood count and bilirubin levels also improved. A month later a repeat blood count demonstrated a recurrent elevation in the reticulocyte count but a normal haemoglobin level, indicating further compensated hemolysis.

There are over 200 known G6PD gene mutations and thus over 200 G6PD variants. Therefore, variants are classified according to clinical severity based on residue G6PD activity in the red blood cell [3,4]. Individuals with class-1 variants have a residual G6PD activity of less than 10% of normal accompanied by chronic non-spherocytic hemolytic anemia or acute exacerbations, class-II variants have similarly low G6PD activity but have no symptoms in the steady state, class-III variants exhibit 10-60% activity with no symptoms in the steady state, and class-IV and class-V variants have 100% and above 100% activity and no symptoms [3,4]. Our patient had a variant in the class-I category, evidenced by a G6PD enzyme activity less than 10%, chronic unconjugated hyperbilirubinemia, a repeat elevation in reticulocyte count (bone marrow response to hemolysis) one month after recovery, and all in the absence of any precipitating factors or stressors.

Several reports indicate that G6PD deficiency confers protection against all forms of malaria, especially in hemizygous deficient males, and heterozygous deficient females, however, results have been conflicting [9-11]. The postulated mechanism is that the uncontrolled oxidative stress in G6PD deficient red cells makes them inhospitable to the malaria parasites [9-11].

There have been several studies looking at the interaction between G6PD deficiency and Gilbert’s syndrome in neonates. Neonates with both conditions appeared to have higher unconjugated bilirubin levels compared to neonates with only G6PD deficiency, possibly due to the inability to conjugate the extra bilirubin load [12,13]. Both conditions are harmless in the steady state, both conditions have precipitating factors, and both produce unconjugated hyperbilirubinemia.

It is important to note that serum bilirubin levels can rise abnormally high in various conditions in individuals who have Gilbert’s syndrome, thereby misleading doctors toward false diagnoses. On the other hand, there are a multitude of other causes of unconjugated hyperbilirubinemia, such that an improper diagnosis of Gilbert’s syndrome can lead to missing a more serious diagnosis [14,15]. The differential diagnosis of unconjugated hyperbilirubinemia includes conditions that cause hemolysis, such as autoimmune hemolysis, sickle cell disease, thalassemia, hereditary spherocytosis, prosthetic heart valves, reabsorption of large hematomas, and drugs (e.g., methyldopa, sulfasalazine, dapsone), and genetic conditions involving severe impairment of bilirubin conjugation, such as Crigler-Najjar syndrome that causes severe hyperbilirubinemia from childhood [15]. The above causes of hemolysis were ruled out in our patient’s case. Additionally, he did not have a history of jaundice during childhood.

Artemisinin drugs (e.g., artemether/lumefantrine) can also cause post-artemisinin delayed hemolysis (PADH) in 15-43% of patients being treated for severe malaria. It typically occurs 7-30 days after initiation of therapy. It is thought that drug-induced death and expulsion of the malaria parasite precipitates early destruction of the previously infected red blood cell [16]. Our patient had elevated reticulocyte counts with normal hemoglobin levels on day-31 and day-55 after antimalarial therapy, indicating underlying compensated hemolysis. Therefore, PADH as a cause of hemolysis cannot be completely ruled out in this case.

This is the first case report examining the exacerbation of unconjugated hyperbilirubinemia during the occurrence of falciparum malaria in a patient who was also discovered to have G6PD deficiency and Gilbert’s syndrome. The exacerbation of hyperbilirubinemia in our patient with chronic mild hyperbilirubinemia could have occurred due to three reasons: (1) the influence of febrile illness and stress in a patient who has Gilbert’s syndrome, (2) the influence of infection in a patient who already has acute-on-chronic spontaneous hemolysis from severe G6PD deficiency on a background of Gilbert’s syndrome, and (3) the influence of malaria parasite-induced red cell hemolysis with resultant unconjugated hyperbilirubinemia on a background of Gilbert’s syndrome. The repeat episode of hemolysis a month after treatment could have been due to an exacerbation of G6PD deficiency and/or contribution from an episode of PADH.

## Conclusions

Unconjugated hyperbilirubinemia is a condition that has a wide differential diagnosis. Gilbert’s syndrome and G6PD deficiency are common genetic disorders that can give rise to unconjugated hyperbilirubinemia, especially during provocation by precipitating factors. Falciparum malaria-induced hemolysis is an infective cause of unconjugated hyperbilirubinemia, and the use of artemisinin combination therapy for uncomplicated falciparum malaria can also cause post-artemisinin delayed hemolysis and unconjugated hyperbilirubinemia. The fact that these four conditions occurred concurrently in the same patient exhibiting unconjugated hyperbilirubinemia, makes this case very interesting. Additionally, this concurrent combination can lead to misinterpretation of the hematological and biochemical markers of hemolysis.
